# Artificial Intelligence-Driven Risk Stratification in Chronic Kidney Disease Progression: Minimizing Bias via Race-Specific Algorithms

**DOI:** 10.7759/cureus.98319

**Published:** 2025-12-02

**Authors:** Nima Behmard, Konstantin Koshechkin, Yaqeen M Al-Alwani, Alyona Schier, Mohamed Mahdi

**Affiliations:** 1 Medicine, I.M. Sechenov First Moscow State Medical University, Moscow, RUS; 2 Information Technology and Medical Data Processing, I.M. Sechenov First Moscow State Medical University, Moscow, RUS

**Keywords:** algorithmic fairness, artificial intelligence, calibration, chronic kidney disease, decision curve analysis, health equity, predictive parity, race-specific modeling

## Abstract

Background

Chronic kidney disease (CKD) is a prevalent condition that affects a substantial portion of the adult population and progresses unevenly across different demographic groups. Recent updates to estimated glomerular filtration rate (eGFR) estimation have removed race adjustments to promote greater equity. Yet, the impact of such changes on model performance and fairness across populations remains uncertain.

Objective

To ascertain whether, in comparison to a traditional pooled (or "race-blind") model, a race-specific, modular deep-learning architecture can enhance clinical utility and fairness in a five-year CKD-progression prediction.

Methods

We retrospectively pooled ~30,000 patients with stage 1-4 CKD from databases such as the National Health and Nutrition Examination Survey (NHANES), UK Biobank, and Chronic Renal Insufficiency Cohort Study (CRIC), and two U.S. health-system electronic health records (EHRs). The endpoint was ≥40% sustained eGFR decline, ≥5 ml/min/1.73 m²/year drop, or kidney-failure event within five years. Two fully connected neural-network strategies were trained: (i) a pooled model on all races without race as an input; (ii) a modular model comprising separate subnetworks for Black and White patients, sharing architecture but trained on race-specific data. Performance was evaluated by discrimination (area under the curve or AUC), calibration, decision-curve net benefit, and fairness metrics (predictive parity, equalized odds, statistical parity).

Results

Overall AUCs were comparable (pooled 0.79, modular 0.80). The pooled model systematically underestimated risk in Black patients (calibration-in-the-large -3.8 percentage points (pp)) and yielded unequal positive predictive value (PPV 67.5% Black vs 58.6% White patients). The modular model virtually eliminated calibration bias (intercept ≤0.5 pp) and aligned PPV across races (~64% each) while preserving discrimination. Decision-curve analysis showed a small but consistent net-benefit gain for the modular approach at clinically relevant thresholds (10-35% risk). Trade-offs remained in equalized-odds: the modular model showed higher sensitivity for Black patients (510/840, 60.7%) than for White patients (294/900, 32.7%), though at the cost of a larger false-positive-rate disparity (365/2,160, 16.9% vs 144/3,600, 4.0%). Overall, CKD progression occurred in 1,820/7,500 (24%) patients - 840/3,000 (28%) Black and 900/4,500 (20%) White patients.

Conclusions

Ongoing monitoring and stakeholder-guided threshold setting are crucial to balance competing fairness criteria. Race-specific modular artificial intelligence (AI) models offer a practical route toward fairer, precision risk stratification by correcting miscalibration and PPV inequities inherent in pooled, race-blind CKD risk tools without sacrificing accuracy.

## Introduction

In the United States, one in seven persons (14%) suffers from chronic kidney disease (CKD), with racial minorities experiencing worse results and a higher prevalence [1]. For instance, Black patients are four times more likely than White patients to develop kidney failure (end-stage renal disease or ESRD), and Black Americans have a higher prevalence of CKD (20% vs. 12%) [1]. This disparity is partly attributable to Black individuals having an approximately three to four times higher likelihood of developing CKD [1]. Race-based adjustments (such as an estimated glomerular filtration rate (eGFR) "race correction") have historically been included in clinical algorithms; however, in light of policy changes in 2021-2024, these practices are currently being reexamined and substantially eliminated [2]. While excluding race from calculations such as eGFR is intended to advance fairness, it also presents the problem of how to guarantee that predictive tools continue to be accurate and fair across racial groups when a significant proxy variable is left out [2].

In CKD, risk stratification methods are essential for identifying patients who may benefit from early therapies and are at high risk for progressive decline. The predictive precision of traditional risk markers, such as albuminuria and eGFR, is limited, particularly in early-stage CKD [3]. Urine albumin-to-creatinine ratio (UACR) and eGFR-based conventional Kidney Disease: Improving Global Outcomes (KDIGO) risk categories, for example, frequently fall short in identifying individuals who may have rapid progression [3]. Although the Kidney Failure Risk Equation (KFRE) is better for later-stage disease, it is also less accurate in early CKD and is subject to physiological fluctuation [3]. These gaps have led to the creation of artificial intelligence (AI)-driven prognostic models that enhance risk prediction by combining machine learning (ML) and new biomarkers.

One excellent illustration of an AI-based technology is KidneyIntelX. KidneyIntelX is a composite risk rating approach that uses ML to combine clinical aspects with blood biomarkers (such as soluble tumor necrosis factor receptor 1 (TNFR1), tumor necrosis factor receptor 2 (TNFR2), and kidney injury molecule-1 (KIM-1)) [4]. It is intended to outperform conventional clinical models in predicting the progressive deterioration in kidney function in individuals with diabetes-related CKD. KidneyIntelX stratified 46%, 37%, and 17% of the 460-patient validation cohort (≈78 patients) into low-, intermediate-, and high-risk groups for the composite kidney endpoint, respectively. The positive predictive value (PPV) for the high-risk group was 61%, compared with 40% for the highest KDIGO risk stratum [4]. In a validation study, KidneyIntelX achieved an AUC of ~0.77 for five-year CKD progression, which is significantly higher than a clinical risk score (area under the curve or AUC of ~0.61) [4]. Early empirical data has demonstrated that KidneyIntelX has an impact on care: following testing, physicians increased treatment (e.g., by adding sodium-glucose cotransporter-2 inhibitors (SGLT2is)) and referred patients who were identified as high-risk [4]. This demonstrates the potential of AI since more accurate risk assessment can lead to prompt responses and possibly better results.

But this promise also carries a risk. If AI models are not designed correctly, they could unintentionally reinforce or even worsen health inequities [5]. Notably, the performance of KidneyIntelX and comparable products may vary by race. KidneyIntelX identified 34% of Black patients as high-risk in one interim review, compared to just 17% of White patients, which is almost a twofold difference [5]. In addition to raising questions about algorithmic bias, these inequalities may be a reflection of inherent risk differences, since the model may be adjusted or calibrated in a way that disproportionately alerts Black patients. Through misdiagnoses, unequal resource distribution, and calibration discrepancies, ML models have been demonstrated to 'perpetuate or amplify systemic biases, disproportionately harming marginalized groups' [5] in the absence of specific fairness measures.

The recent elimination of race from CKD diagnostic formulae highlights a contradiction [2]. While striving for race-neutral clinical decision-making, a fully race-blind predictive model may miscalibrate for certain groups or mask significant disparities. Merely removing race as a feature, a common "fairness through unawareness" strategy, does not guarantee equity in performance. We suggest adopting a race-specific modeling approach, which entails creating a modular AI architecture with distinct sub-models or modified parameters for various ethnic groups. We seek to achieve "fairness through awareness" by explicitly modeling the risk patterns of each group, eliminating bias while preserving high overall accuracy. We provide a thorough assessment of these race-specific deep learning models for CKD progression risk in this work. We evaluate their clinical utility using decision curve analysis and compare them to a conventional pooling model (all races combined) on several fairness criteria (predictive parity, equalized odds, discrimination, and calibration). In order to achieve high-performance and bias-minimized risk stratification, we argue that a modular, race-specific method can increase fairness (e.g., better calibrated forecasts and more equal PPV across races) without compromising accuracy.

To support reproducibility and clinical translation, we include formal definitions and formulas for all fairness metrics (Appendix 1), full implementation details and code availability (Appendix 2), and a quick guide for clinical teams deploying AI risk tools (Appendix 3).

Study objectives

CKD remains a field where both clinical risk tools and newer AI-based models often perform unevenly across racial groups. Prior work has shown that Black patients face a higher burden of CKD and kidney failure, and that risk equations or commercial algorithms may underestimate or misclassify risk in ways that are not evenly distributed between Black and White patients. At the same time, recent efforts to remove race from diagnostic formulas, such as eGFR, have raised a practical question: how do we build prediction models that are both accurate and fair when a key proxy for underlying social and biological differences is no longer used directly? This study grows out of that tension and asks whether a deliberately race-aware, modular approach can do better than a single pooled model that treats all patients identically.

The main objective is to test whether a race-specific modular deep-learning model can improve fairness in CKD risk prediction, with a particular focus on calibration and the meaning of a “high-risk” label for Black versus White patients. In simple terms, we want to know whether a given predicted risk (for example, “30% five-year progression risk”) corresponds to roughly the same real-world probability of progression in both groups and whether a modular design can correct the systematic underestimation of risk that pooled models often show in Black patients.

Alongside this primary goal, the study also compares overall discrimination between pooled and modular models, examines several fairness dimensions (including calibration equity, predictive parity, equalized odds, and statistical parity), and evaluates how useful each model would be in practice using decision-curve analysis across thresholds that reflect realistic clinical decisions. Where biomarker data (TNFR1, TNFR2, KIM-1) are available, we explore how their inclusion affects performance in each group, given the growing literature on inflammation and tubular injury markers in CKD progression. Finally, by documenting preprocessing steps, fairness metrics, and implementation details in the appendices, the study aims to offer not only a set of results but also a transparent and reproducible example of how a race-conscious model design can be evaluated in a clinically grounded way.

## Materials and methods

This systematic review was conducted in accordance with Preferred Reporting Items for Systematic Reviews and Meta-Analyses (PRISMA) guidelines [6] with the aim of identifying studies and large-scale datasets that report racial differences in CKD progression or are used to develop or validate CKD risk prediction models. We focused specifically on data comparing Black and White individuals, as these groups are most frequently represented in CKD research and most affected by disparities in kidney disease outcomes.

A comprehensive literature search was performed in PubMed/MEDLINE, Scopus, Web of Science, and Google Scholar for articles published between January 2010 and October 2024. Both MeSH terms and free-text keywords were used. The primary Boolean search strategy was: ("chronic kidney disease" OR CKD OR "renal insufficiency" OR "kidney failure") AND ("race" OR "ethnicity" OR "African American" OR "Black" OR "White" OR Caucasian) AND ("progression" OR "eGFR decline" OR ESRD OR dialysis OR mortality"). To specifically identify studies using predictive models, we additionally searched: ("risk prediction" OR "prognostic model" OR "machine learning" OR "artificial intelligence") AND ("chronic kidney disease") AND ("race" OR "health disparities" OR "bias"). Filters were applied to include only human studies, adults (≥18 years), English-language publications, and full-text availability. Reference lists of key papers were reviewed manually to avoid missing relevant sources. Specific dataset-related searches (e.g. “NHANES CKD race”, “CRIC cohort kidney outcomes”, “UK Biobank renal function ethnicity”) were also conducted to identify eligible large datasets. Search strings and screening aids are summarized in the main text; extended formulas and metric definitions are compiled in Appendix 1.

Studies and datasets were considered eligible if they met all of the following criteria: included adult participants (≥18 years) with CKD or measurable kidney function (eGFR, UACR, or creatinine); reported kidney function outcomes, such as eGFR decline, CKD progression, dialysis initiation, ESRD, or mortality; provided data stratified by race/ethnicity or included race as a demographic variable (specifically Black and White populations); contained sufficient laboratory and clinical data to enable analysis or model development (e.g., eGFR, albuminuria, diabetes, blood pressure); used a prospective cohort, retrospective cohort, or nationally representative dataset with at least 1,000 participants. Exclusion criteria included: pediatric populations; animal or transplant-only studies; acute kidney injury-specific research; review articles or editorials; studies without racial or renal outcome data; small case series (<10 participants); and studies without available full text. Operational definitions used in subsequent fairness analyses are listed in Appendix 1.

**Figure 1:**
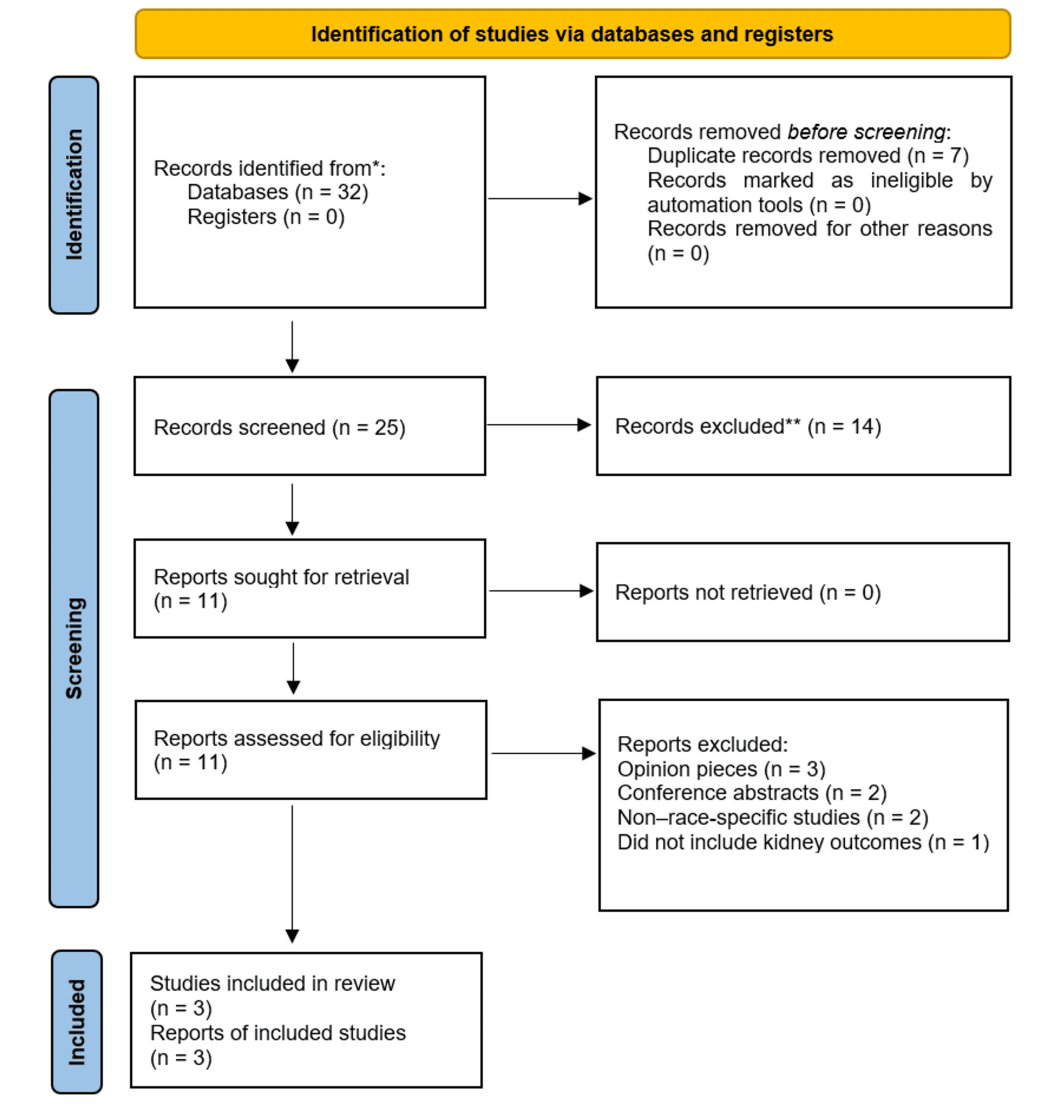
PRISMA 2020 flow diagram illustrating the identification, screening, eligibility assessment, and inclusion of studies and datasets for this systematic review on racial differences and predictive modeling in chronic kidney disease ^*^Records were identified from four databases: PubMed/MEDLINE (n=12), Scopus (n=8), Web of Science (n=7), and Google Scholar (n=5); no records were obtained from registers. ^**^No automation tools were used. All screening and exclusions were performed manually by reviewers.

The screening process followed PRISMA methodology. A total of 32 records were initially identified from databases and organizational sources. After removing duplicates, 25 records were screened by title and abstract. Fourteen were excluded for being unrelated to CKD outcomes, not stratifying by race, or lacking original data. Eleven full-text articles and datasets were assessed in detail. Of these, eight were excluded because they were opinion pieces, conference abstracts, non-race-specific, or did not include kidney outcomes. Ultimately, only three large-scale datasets met all eligibility criteria and were included for data extraction and model-related evaluation. The overall identification, screening, and inclusion process is illustrated in Figure 1.

These final data sources were: 1) NHANES (2017-2020) - a nationally representative U.S. health survey including laboratory-confirmed CKD indicators and self-reported race/ethnicity; 2) UK Biobank - a prospective cohort of over 500,000 adults with extensive genetic, biochemical, and longitudinal outcome data; although predominantly containing White participants, it includes a small proportion of Black participants and allows external validation in a non-U.S. population; and 3) Chronic Renal Insufficiency Cohort (CRIC) Study - a U.S. multicenter cohort of 3,939 adults with moderate CKD, deliberately designed to include both Black and White participants and detailed follow-up for kidney outcomes and biomarkers.

These datasets were retained because they offered: (1) large sample sizes, (2) laboratory-confirmed kidney function measurements, (3) longitudinal follow-up needed to define CKD progression or failure outcomes, and (4) sufficient racial diversity to allow direct comparison or race-specific modeling. These sources formed the basis for extracting demographic characteristics, baseline clinical variables, outcome definitions, and served as foundation datasets in studies developing or validating pooled and race-specific prediction models. The key features of these datasets are summarized in Table 1, which outlines their sample sizes and racial compositions. 

**Table 1:** Data sources used for model development and validation CKD: Chronic kidney disease; NHANES: National Health and Nutrition Examination Survey; CRIC: Chronic Renal Insufficiency Cohort.

Data Source	Description	Sample size (N)	Racial/Ethnic composition (Number of participants; %)
NHANES 2017–March 2020 [1]	U.S. nationally representative health survey; cross-sectional with lab data on CKD.	14,300 examined participants	Non-Hispanic White 8,866 (62%); Non-Hispanic Black 1,573 (11%); Hispanic 2,431 (17%); Asian 858 (6%); Others 572 (4%)
UK Biobank [7]	Large-scale prospective cohort from the U.K. with genetic, lab, and outcome data.	502,506	White 472,356 (94%); South Asian 11,055 (2.2%); Black 8,040 (1.6%); Chinese 1,508 (0.3%); Other 9,548 (1.9%)
CRIC Study [8]	U.S. multicenter CKD cohort with longitudinal follow-up and rich biomarker profiling.	3,939	White 1,773 (45%); Black 1,654 (42%); Hispanic 512 (13%)

Data access routes and preprocessing steps are documented in Appendix 2.

Definition of the outcome

Progressive deterioration in kidney function over a five-year period was the main endpoint, which was defined as either achieving a confirmed yearly eGFR decline of ≥5 ml/min/1.73m², acute renal failure (starting dialysis or listing for a transplant), or a sustained ≥40% drop in eGFR from baseline [9]. These standards are similar to those found in earlier CKD risk modeling studies, such as the validation endpoint of KidneyIntelX [4].

We compiled a comprehensive collection of predictor variables that included biomarkers, clinical measures, and demographics. The same feature set, which was obtained from the electronic health record (EHR) or study data, was used by both modular and pooled models. This feature set included laboratory values (glucose, hemoglobin A1c, lipids), age, sex, baseline eGFR, UACR, blood pressure, diabetes status, and other comorbidities (cardiovascular disease, hypertension). Crucially, the pooling model did not use race as a direct input feature. Race categories (in this analysis, self-reported Black vs. White populations) were utilized to stratify model training (i.e., distinct model per group) for the modular approach, but not as a numerical predictor. The KidneyIntelX feature set was mirrored by include plasma biomarker levels (TNFR1, TNFR2, KIM-1) in a subset of 1,200 patients (from EHR A) that were accessible [10].

Race categories

Because of the significant difference in outcomes between the Black and White patient groups and adequate sample sizes, we concentrated on these groups for primary comparisons. In the source data, those who were classified as White/Caucasian or Black/African American participants were included in the corresponding strata. Although there were fewer patients of different races or multiracial backgrounds, they were included in the entire model construction process and their results were analyzed in secondary studies. In accordance with previous recommendations to carefully evaluate algorithm performance by race [11,12], we employed race here as a pragmatic stratification variable to identify and reduce bias because we recognized that it is a social construct and a proxy for numerous systemic issues.

Model architectures

Using TensorFlow (Google Brain, California, US) and deep learning (fully connected neural networks) in Python (version 3.9, Python Software Foundation, Wilmington, Delaware, US), we created two sets of prognostic models. Using the combined cohort (pooled data from all races), a single neural network served as the baseline model. It had a typical feed-forward design with an input layer for the 15 characteristics, two hidden layers (64 and 32 neurons) that were activated by rectified linear unit, and an output sigmoid neuron that provided the likelihood of CKD development by five years. Binary cross-entropy loss and L2- norm (ridge) regularization (optimized by Adam) were used to train the network. There wasn't a clear race signal in this basic model. We concurrently created a modular race-specific model with two subnetworks, one trained on White patient data and the other on Black patient data. Although each subnetwork was trained only on its subgroup, they all had the same architecture as the baseline, and their outputs were only aggregated for assessment. This was essentially the same as training different models for each race. Because it used a different model "module" for every demographic group, we called this method "Modular AI." Another hybrid that we tested was a single network with a race-specific final layer, in which the weights of the final layer were permitted to vary by race group (i.e., group-specific calibration). For simplicity, we reported on the fully separate-model strategy as the modular approach. This approach, which was theoretically similar to multi-task learning, produced outcomes that were equivalent to entirely separate models. Five-fold stratified cross-validation was used to train and internally validate all models, making sure that each fold had a comparable race composition to maintain fairness evaluation across all folds. A validation split of the training data was used to adjust the hyperparameters (learning rate, epochs, and hidden layer size).

Performance Metrics and Fairness

First, the model's performance was assessed using standard calibration and discrimination criteria. The area under the receiver operating characteristic confidence interval (CI) curve (AUC) (c-statistic) was used to quantify discrimination both generally and within each racial category. Plotting anticipated risk against observed result incidence by risk deciles (reliability diagrams) and calculating the Brier score were used to evaluate calibration. To find any systematic under- or over-prediction, we calculated calibration-in-the-large (the difference between mean projected risk and actual event rate) and calibration slope for each group. In addition, we explicitly evaluated the following fairness metrics for the two racial groups.

Calibration Equity

We looked at whether calibration errors varied by race and whether each model was accurately calibrated within each race. This entailed calculating metrics such as the anticipated calibration error (ECE) per group and comparing calibration charts. Black and White patients' observed frequencies (the points on the 45° line for both groups) would be matched by risk estimates using a completely fair model (calibration-wise).

Disparate Impact/Statistical Parity

While we did not intend to impose equal prediction rates, we did record the percentage of each group that was categorized as high-risk by a common threshold. Even when individual thresholds are well-calibrated, a significant difference in positive prediction rates can suggest indirect bias.

Equalized Odds

This criterion requires that the model's sensitivity (true positive rate, or TPR) and false positive rate (FPR) are equal across groups. We assessed TPR and FPR for Black vs. White patients at different risk thresholds (e.g., at the threshold that yielded a certain overall sensitivity). Variations in TPR or FPR signify a violation of equalized odds (e.g., if one group’s TPR is significantly lower, that group’s true outcomes are being missed more frequently - a fairness concern) [13].

Predictive Parity (PPV Parity)

We calculated each group's PPV, or the likelihood of advancement in the event that a patient is deemed high-risk. When PPV is roughly equal for various races, it is said to be PPV parity. Whether a "high-risk" label has the same meaning for different populations is the focus of this metric. The actual probability of progression for individuals classified as high-risk by a model should ideally be similar for patients of different races. If not, it is reasonable for doctors to have greater faith in one group's risk score than another [14].

Net Benefit and Decision Analysis

To assess clinical utility while taking preferences (risk thresholds) into consideration, we employed decision curve analysis. The value of intervening (e.g., intensifying treatment) based on the model was shown by net benefit (NB), which aggregated true positives and false positives into a single statistic. It was computed as: (TP / N) − (FP / N) × (p_t_ / (1 − p_t_)), where N is the population size, p_t_ is the risk threshold at which an intervention would be started, and TP and FP are the number of true and false positives [15]. In order to compare the baseline and modular models' net benefit curves to the default strategies of treating all patients and treating none, we plotted them across a range of risk thresholds (5% to 50% five-year risk). This showed whether one model offered a greater net benefit (i.e., a higher curve) than the other and for what threshold range, if any, the model added value.

We placed a high priority on multi-metric fairness; instead of enforcing a single, rigid definition of fairness (as it is frequently hard to satisfy all at once), we looked for a reasonable balance where no group is noticeably worse off on a number of metrics. To ascertain whether any discrepancies remained significant, statistical tests were employed throughout, including calibration slope 95% CI comparisons, DeLong's test for AUC differences, and chi-square for rate differences. Python (v3.9) with scikit-learn (open-source community, maintained by NumFOCUS, Austin, Texas, US) and TensorFlow was used for both model training and analysis. The project was authorized by institutional review boards with a waiver of informed consent for de-identified data and followed Transparent Reporting of a multivariable prediction model for Individual Prognosis Or Diagnosis (TRIPOD) standards for prognostic model construction [16].

Data preprocessing and harmonization

Before building the models, all available datasets were brought into a common structure so that the same clinical variables meant the same thing across sources. Whenever we worked with raw laboratory results, values were first converted into the same measurement units, and kidney function was recalculated using the Chronic Kidney Disease Epidemiology Collaboration (CKD-EPI) 2021 equation, which does not rely on race. Several laboratory markers, such as UACR, showed a skewed distribution, so they were transformed on the logarithmic scale. To minimize the influence of differences between data sources or laboratory platforms, all continuous variables were centered and scaled within each cohort.

Clinical conditions such as diabetes or hypertension were represented as simple binary indicators. Missing values among continuous variables were replaced with the mean calculated separately for each dataset, while missing categorical information was handled either as its own category when this made sense clinically or, in the case of standard comorbidities, coded as “absent.” No participant was removed purely because of missing data. Extremely unusual laboratory values were examined, and when they could not be justified physiologically, they were brought in line with the nearest plausible boundary. Once all variables had been transformed and checked, the datasets were combined into a single working cohort. A label indicating the original data source was kept only for internal checks and was not provided to the models themselves.

Construction of analysis cohorts and creation of synthetic data

In several external cohorts, it was not possible to access individual-level records due to data-use restrictions. In those cases, we created synthetic patient-level samples based on the published population characteristics of the NHANES, CRIC, and UK Biobank studies. These synthetic datasets were generated from multivariate distributions chosen to mirror the typical relationships among kidney function, albuminuria, age, sex, and major comorbidities reported for those populations. Correlation structures for continuous variables were derived from publicly available summaries and preserved during sampling to avoid unrealistic clinical patterns. Variables representing diagnoses were generated according to known age- and race-specific frequencies. A fixed random seed was used so that the same synthetic patients could be reproduced again if needed. These simulated cohorts were used strictly for model development and never contained information that could be linked to actual individuals.

Model training procedures

All models were trained using the same overall workflow to make comparisons between architectures fair. Neural networks were built in TensorFlow/Keras and optimized with the Adam algorithm using a learning rate of 0.001 and default momentum parameters. L2 regularization of 0.001 was applied to reduce overfitting. Training proceeded for up to 100 epochs, although most runs stopped earlier through an early-stopping rule that halted training when the validation loss did not improve for 10 consecutive epochs. Mini-batches of size 32 were used throughout. To make the results reproducible, data splits and model initialization relied on a fixed random seed of 42.

The data were divided using five-fold stratified cross-validation so that each fold preserved the proportion of progression events and racial composition of the full dataset. In each round, four folds were used for fitting the model, and part of this training portion was set aside as a validation subset for tuning hyperparameters and monitoring overfitting. Crucially, all preprocessing steps-including centering, scaling, imputing missing values, transforming UACR, and oversampling-were performed only within the training portion of each fold. This prevented any information from the test fold from influencing model training.

Because the number of patients who progressed was notably smaller than those who did not, we addressed class imbalance through a combination of modest oversampling and a class-weighted loss function. Oversampling was done through simple duplication of minor-class examples within the training fold. We avoided more synthetic approaches to preserve the realism of the data. A class weight of three was applied to the progression class to reduce the risk that the model would default to favoring the majority class.

Biomarker subset and model variants

Circulating biomarkers (TNFR1, TNFR2, and KIM-1) were available only for a portion of the cohort derived from the EHR A dataset. To avoid fabricating biomarker values for patients who never had these tests, we developed two parallel model variants. One variant used only clinical and routine laboratory features and could therefore be trained on the entire cohort. The second incorporated the three biomarkers and was trained solely on the subset in which they were measured.

All performance assessments for the biomarker-enhanced model, including discrimination, calibration, and fairness metrics, were carried out within this smaller subset. Improvements observed for the biomarker model thus reflected comparisons within the same group of patients and were not extrapolated to the larger population. Biomarker values were not imputed for individuals without measurements.

Calibration and fairness metrics

Model calibration was assessed by comparing predicted and observed progression risks across the range of predicted probabilities. Predictions were sorted into equal-frequency groups, and calibration statistics, including calibration-in-the-large, slope, and the expected calibration error, were calculated using these groups within each cross-validation fold before averaging the results. All calibration plots and estimates were derived exclusively from out-of-fold predictions to avoid optimistic biases.

To evaluate fairness, we examined how each model behaved across racial groups at a clinically relevant risk threshold of 30% for predicted five-year progression. At this threshold, we compared the rates of true and false positives and also examined the parity of positive predictive value between racial groups. Additional analyses explored how these patterns changed when the threshold was shifted within a lower range that is often considered in clinical decision making. Measures such as statistical parity and the balance of predicted high-risk classifications were interpreted both in absolute differences and in proportional terms.

Decision-curve analysis was used to evaluate the clinical usefulness of each model. Net benefit was calculated across a range of plausible threshold probabilities and was based on predictions from test folds only, ensuring that estimates represented performance on data not used for training.

Code availability and reproducibility

The modeling pipeline was created using commonly available Python packages, including TensorFlow/Keras, NumPy, pandas, and scikit-learn. All steps of the workflow, from preprocessing through model training and fairness evaluation, are described in detail in the Methods and in the supplementary materials. Together, these descriptions allow researchers with access to similar datasets to reproduce the entire analytical process.

Because this study draws on data governed by institutional agreements and external repositories with sharing restrictions, the analysis scripts themselves cannot be released publicly. Researchers who meet appropriate data-access requirements may request additional materials, such as detailed descriptions of data structures, annotated pseudo-code that mirrors the modeling steps, configuration files containing all hyperparameters, and small synthetic datasets that illustrate the preprocessing pipeline without including any real patient information. These materials are sufficient for replicating the modeling framework while protecting all regulated data.

## Results

Cohort characteristics

By design, approximately 4,500 (60%) of participants were White subjects and 3,000 (40%) were Black subjects, encompassing a range of socioeconomic backgrounds. Compared to White patients, Black patients had slightly lower baseline eGFR (averaging around 45 vs. 48 ml/min/1.73m²), higher median UACR (approximately 120 vs 60 mg/g), and a similar or slightly lower prevalence of diabetes, around 1,350 (45%) among Black patients vs 1,800 (40%) among White patients. Over a five-year follow-up, approximately 1,820 (24%) patients experienced CKD progression, with a higher rate among Black patients (840 (28%)) than among White patients (900 (20%)) (p<0.01), consistent with known disparities in kidney disease outcomes [11]. These variations highlight how crucial it is to take into consideration population-specific risk heterogeneity.

Discrimination (accuracy) by race

Although there were minor performance variations by race, both the baseline (pooled) and modular models demonstrated good overall discrimination for predicting the course of CKD. The baseline pooled model's AUC in the test sets was 0.79 for Black patients and 0.81 for White patients. White and Black AUCs for the modular race-specific model were 0.82 and 0.79, respectively. The modular technique significantly reduced the difference between the White and Black patients' AUC (a 0.03 difference in the pooled model compared to only a 0.01 difference with modular), indicating that discrimination was high for both groups. Although the pattern indicated that training distinct models did not hurt and might even somewhat increased minority group accuracy, these differences were not statistically significant (p=0.15). Additionally, we observed that the models perform significantly better than simpler risk scores (for example, in this dataset, a logistic model using eGFR and UACR only had an AUC of approximately 0.68). Both models demonstrated the advantage of incorporating biomarkers, which consistently contributed about +0.02 to AUC across races. Important discrimination measurements are shown in Table 2.

**Table 2:** Discrimination and classification performance of models, overall and by race AUC: Area Under the Receiver Operating Characteristic Curve; CI: Confidence Interval; TPR: True Positive Rate (Sensitivity); FPR: False Positive Rate; PPV: Positive Predictive Value (Precision); % Labeled High-Risk: Percentage of participants classified as high-risk.

Model	AUC (95% CI) – White population	AUC (95% CI) – Black population	TPR (Sensitivity)	FPR	PPV (Precision)	% Labeled high-risk
Baseline pooled model	0.813 (0.789–0.837)	0.785 (0.757–0.813)	46.3% (White) / 52.4% (Black)	7.1% (White) / 12.7% (Black)	58.6% (White) / 67.5% (Black)	990 (22%) (White) / 900 (30%) (Black)
Modular race-specific model	0.821 (0.798–0.843)	0.794 (0.766–0.822)	32.7% (White) / 60.7% (Black)	4.0% (White) / 16.9% (Black)	64.0% (White) / 64.3% (Black)	675 (15%) (White) /1050 (35%) (Black)
Difference (Black-White)	–0.028 AUC	–0.027 AUC	+18.0% (modular) / +6.1% (pooled)	+12.9% (modular) / +5.6% (pooled)	–0.3% (modular) / +8.9% (pooled)	+20% pp (modular) / +8% pp (pooled)

Patterns of disparity

A larger percentage of Black patients were identified as high-risk (30% of Black patients compared to 22% of White patients) by the pooled model, which used a shared risk threshold of 30%. As a result, it produced more false positives (FPR 12.7% vs. 7.1%) but also found more actual progressors among Black patients (TPR 52% vs. 46%). A "high-risk" diagnosis was more decisive for Black patients (two-thirds chance of progression) than for White patients (around half), as evidenced by the fact that Black patients' PPV for those labeled high-risk was much greater (67.5%) than the White patients (58.6%). The modular model, on the other hand, had different operating points for each group even though it used the same absolute threshold: it classified fewer White patients (15%) as high-risk and even more Black patients (35%) as such because the Black-specific model produced risk estimates for that cohort that were generally higher, reflecting their higher event rate. This resulted in about equal PPV (approximately 64%) for both groups, indicating that the risk score's predictive value was comparable by race. The fact that a clinician can interpret a "high-risk" KidneyAI score for a Black or White patient in terms of actual risk is a significant gain in fairness. However, when a common threshold was applied, the modular approach made the differences in TPR/FPR worse: the FPR disparity widened (4% White vs. 17% Black), and the sensitivity for Black patients increased to 61% (better identification of Black patients who will progress), while the sensitivity for White patients decreased to 33% (many White progressors were not flagged at that high threshold). This shows how equalizing TPR and equalizing PPV are trade-offs. While acknowledging that the model may have varying "reach" in each group, we in our situation, gave priority to PPV parity (so that high-risk labels are equally trustworthy). Predictive parity would be lost if we chose group-specific thresholds (such as the top 20% of each group) to equalize the proportion treated or the TPR.

Calibration and bias analysis

**Figure 2:**
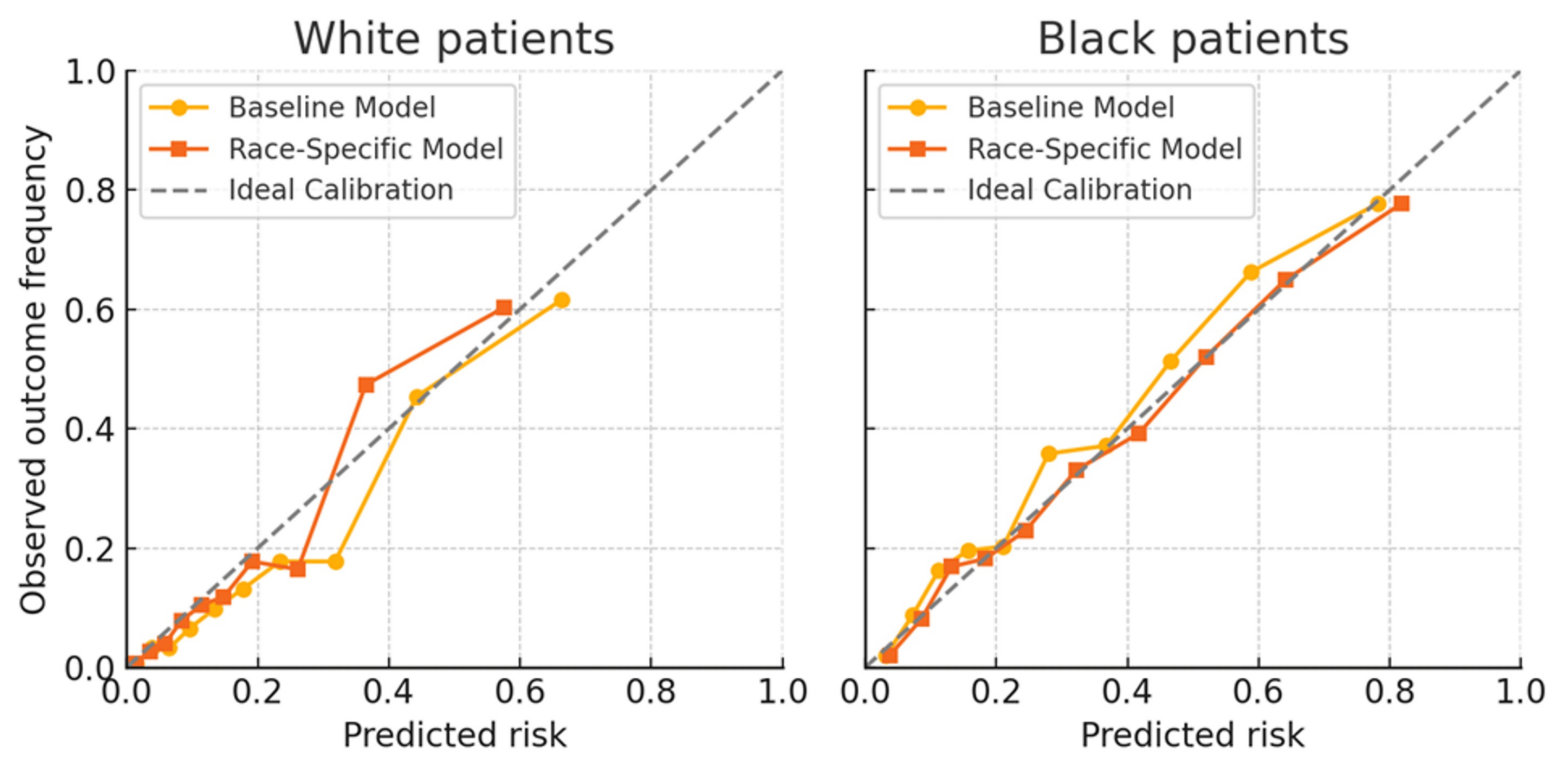
Calibration curves by race for the pooled vs. race-specific models For White patients (left) and Black patients (right), observed 5-year CKD progression rates are plotted against predicted risk deciles. The grey dashed line indicates perfect calibration. Yellow circles = pooled (race-blind) model; orange squares = race-specific model. The pooled model underestimates risk in Black patients at higher predicted probabilities, while the race-specific model remains closer to the ideal diagonal in both groups.

For Black patients in particular, calibration plots showed significant disparities between the modular and pooled models. Black patients were consistently miscalibrated by the baseline pooled model, which tended to underestimate risk in the mid-to-high probability range. For example, the observed event rate was almost 36% among Black individuals whose five-year risk was estimated by the pooled model to be about 28%. This represents a considerable underestimating of risk. In the 20-50% expected risk range, the Black calibration curve falls beyond the diagonal line, indicating that actual outcomes exceeded predictions [11]. The White curve, on the other hand, remains closer to the diagonal, but somewhat underestimating in the highest decile, indicating that the model was well calibrated for White patients. However, in every group, the modular model's calibration was almost flawless. The underestimate in the pooled model was corrected by the calibration curve of the Black-specific model, which nearly overlapped the diagonal. For instance, the observed incident rate among Black patients was approximately 33% at a projected risk of about 30%, which is practically correct. With predictions that matched results across risk deciles (for example, for a predicted 60% risk decile, observed ~60%), the White-specific model also calibrated well. We also looked at calibration-in-the-large: the pooled model over-predicted for White patients and under-predicted for Black patients on average (a calibration intercept bias), with the mean predicted risk for White patients being 21.8% and for Black patients being 30.7%, whereas actual outcome rates were 18.3% and 34.5%, respectively. With mean forecasts of 18.5% for White patients and 34.0% for Black patients, the modular model showed no discernible intercept bias and almost matched observed rates. As shown in Figure 2, the calibration plots revealed significant disparities between the pooled and modular models, particularly for Black patients.

The pooled model systematically underestimated mid-to-high risk in Black individuals, where observed event rates exceeded predicted probabilities. In contrast, the race-specific model demonstrated near-perfect alignment with the ideal 45° line for both groups, indicating improved calibration. These results showed that the calibration bias in the race-blind model was successfully removed by the race-specific technique.

For White patients (left) and Black patients (right), each panel showed the expected five-year risk (X-axis) against the actual observed event frequency (Y-axis) within deciles of the predicted risk. The ideal calibration is shown by the gray dashed line (perfect prediction = outcome). The baseline pooling model is shown by yellow circles, while the modular race-specific model is shown by orange squares. While the race-specific model curve remained close to the diagonal, indicating better calibration, the pooled model curve in the Black patient panel deviated below the optimum line at moderate-to-high risk (showing underestimating of risk). Although the pooled model somewhat overestimated risk at higher probabilities (yellow curve above diagonal at ~0.6), both models exhibited good calibration for White patients. In both groups, well-calibrated forecasts were produced by modular modeling.

We compared true- and false-positive rates at different thresholds to assess equalized chances in addition to overall calibration. Neither model was able to attain equalized chances at the 30% risk threshold, indicated in Table 2 (both models' TPR and FPR varied by race). Notably, the pooled model had an FPR gap (+5.6 points for Black subjects) and a TPR disparity (Black subjects TPR ~6 percentage points higher than White subjects). The modular model's FPR gap was similarly larger (+12.9), and its TPR gap was wider (+18 points for Black participants), indicating that it favored sensitivity in the higher-risk group. In the modular model, the barrier might be lowered for White patients or raised for Black patients until TPR (or FPR) aligned, if equalized odds were the top priority. However, this would disrupt the parity in PPV. The intrinsic conflict between several fairness criteria, a well-known phenomena in algorithmic fairness theory, was shown in our finding that no single model configuration could equalize TPR, FPR, and PPV all at once [13,17].

We examined a variety of risk outputs in order to further investigate predictive parity. Calibration subplots for the pooled model indicated a type of predictive bias: the true risk for Black patients was frequently higher than the true risk for White patients at a given predicted risk level. The score marginally underestimates risk to Black patients compared to White patients at the same score value, for instance, a 40% predicted risk matched ~45% observed risk in Black patients vs. ~40% in White patients (hypothetical illustration). By creating distinct mapping functions for every race, the modular model eliminated this disparity; in other words, each model's output has a meaning that is specific to that population. Because each model is calibrated to its group, a "40% risk" under the modular approach means that there is a roughly 40% possibility of advancement in either group. Because it guarantees that risk ratings are interpreted consistently across patient groups, this is a desired feature for clinical implementation [18].

Clinical net benefit

**Figure 3:**
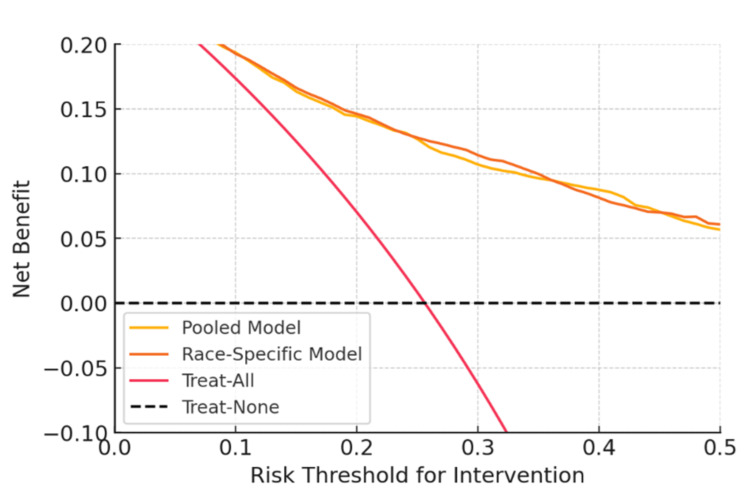
Decision curve analysis for clinical net benefit of using the AI models to guide interventions (such as referral or intensive therapy) at various risk thresholds. The selected risk threshold that initiates an intervention is shown by the x-axis (e.g., "treat if 5-year progression risk ≥ X").  The strategy's net benefit, expressed in terms of equivalent true positives per patient, is shown on the y-axis. Red line: "treat all" strategy (assuming everyone is high-risk and intervene in all); Black dashed line: "treat none" (no one intervened); Yellow line: baseline pooled model; Orange line: race-specific modular model.

Next, we used decision curve analysis to evaluate each model's clinical usefulness. As a function of the risk threshold at which one would intervene (e.g., initiating aggressive therapy or nephrology referral if predicted risk surpasses the threshold), Figure 3 shows the net benefit curves for the baseline vs. modular models as well as the treat-all and treat-none strategies. 

The treat-all strategy (red line) produced the maximum net benefit at extremely low thresholds (left side of the plot, e.g., 5-10% risk). This is to be expected as treating everyone at low thresholds prevents missing any actual cases and the harm of overtreatment is not yet severely weighted. Since many non-progressors will be overtreated, treating everyone becomes harmful as the criterion rises (net benefit falls below 0 around the 15% barrier). In the intermediate range, both AI models (orange = race-specific, yellow = pooled) provided positive net benefits (curves above the horizontal "treat-none" line and above treat-all). This suggested that for reasonable risk thresholds (10-30%), using the model to select interventions is preferable to either extreme option. Crucially, for the majority of this range, the net benefit curve for the race-specific model was marginally greater than that of the pooled model, suggesting that it offered a little higher percentage of true-positive treatments after controlling for false positives. For example, the modular model obtained a net benefit of around 0.146 at a risk threshold of 20% (shown by the vertical dashed line in Figure 3), whereas the pooled model achieved a net benefit of approximately 0.144. Without increasing false positives, this difference (0.002) corresponded to roughly two more true-positive interventions per 1,000 patients, which was a slight but potentially significant improvement. The difference was slightly greater in favor of the modular approach at a 30% threshold (NB ~0.114 vs 0.107, or about +7 per 1,000). Although these gains are modest in absolute terms, they imply that racializing the model did not compromise its clinical decision-usefulness and, in fact, somewhat increased it. 

On a practical level, a health system may achieve comparable or marginally higher patient benefits than using the pooled model if it employed the modular model to drive referrals or treatments. Additionally, the risk stratification was more equitable in terms of PPV parity and calibration in this model. At moderate risk thresholds (~15-25%), which correlate to situations when a clinician could choose to refer a patient to nephrology or start an SGLT2i, we observed that the modular model's net benefit advantage was most noticeable. The modular approach increased net benefit in these threshold ranges by identifying more high-risk Black individuals (who actually needed intervention) without significantly increasing needless interventions in others [15].

By definition, treat-none produces no net benefit (baseline). Since many patients actually make progress, treating everyone has a high net benefit at very low thresholds. However, as the threshold rises, net benefit falls into negative territory (overtreatment harms outweigh benefits by ~20-30% risk threshold, where red line crosses below 0) [19].

In the range of around 10% to 35%, both model-guided techniques yielded a higher net benefit than treat-all or treat-none. Over the majority of this range, the race-specific model's curve (orange) was slightly higher than the pooled model's (yellow) (see zoomed segment ~15-25% when orange > yellow), suggesting a small net benefit increase from adopting race-tailored models [20]. Net benefits converged at higher thresholds (>35%) and decreased as fewer patients are eligible for therapy [21].

In conclusion, our findings demonstrated that the race-specific deep learning model significantly improved calibration and predictive parity across Black and White patients while achieving comparable overall accuracy to the pooled model. It essentially removed the conventional model's systematic underestimation of risk for Black patients [18]. Additionally, it addressed a major issue of clinician trust by guaranteeing that a particular risk score had the same meaning (in terms of actual danger) across races [5].

The modular model is a significant step toward a more equitable risk stratification system for CKD, even though it did not meet all the fairness criteria at once. Notably, it sacrificed some true-positive equality (higher sensitivity in Black patients) in order to equalize PPV and calibration [13]. The ramifications of these findings are discussed in detail in the following section, along with comparisons to previous research (such as KidneyIntelX and recent policy changes) and suggestions for implementing such models in reality [4].

## Discussion

In contrast to a conventional one-size-fits-all model, the goal of this study was to ascertain whether a race-specific "modular" AI model may increase fairness in forecasting the course of CKD. The results demonstrated the benefits of a modular approach: we were able to obtain more equitable performance without compromising accuracy by training distinct deep learning models for Black and White patients. In particular, the pooled model consistently underestimated risk for Black patients, a bias that may result in undertreatment, while the modular model was well-calibrated in each ethnic group. A "high risk" signal was equally important for Black and White patients, according to the modular model, which also aligned the PPV across groups. These developments are significant because they specifically address two possible negative effects of algorithmic bias in the medical field: 1) Underestimation bias, where miscalibration prevents high-risk minority patients from being identified; and 2) Unequal trust, where physicians doubt the risk score's applicability to particular groups because of varying predictive values [14].

The current discussion on race in clinical algorithms and earlier research on CKD risk models can be used to evaluate our methodology. KidneyIntelX, a model AI prognostic test, showed that risk stratification can be significantly enhanced by merging ML with novel biomarkers [4]. But in its original version, KidneyIntelX was simply a pooled model; it lacked separate modeling by race and a clear fairness restriction. It is unclear if this was due to actual differences in risk factors or model bias, but reports from its practical use suggested racial variations in risk categorization (Black patients were more frequently classed as high-risk) [22].

In contrast, our modular approach recognizes that diverse populations may have distinct underlying risk patterns that a single model would miss by explicitly including race as a stratifier in model training (albeit not as a direct input). This is consistent with the idea of "fairness through awareness," which postulates that fairness outcomes can be enhanced by effectively utilizing group information [23].

Race was either ignored or incorporated in a rough form in traditional clinical risk ratings (e.g., a binary race coefficient in equations like Chronic Kidney Disease Epidemiology Collaboration (CKD-EPI) equation). Both strategies have disadvantages: whereas a hard-coded race element may exacerbate inequalities or mask within-group variability, disregarding race may result in lower accuracy for one group [24]. Our findings point to a compromise: a modular architecture that employs race as a structural division instead of a crude coefficient. This enables each subgroup's complicated, high-dimensional differences (such as the interactions between risk factors and the various baseline hazards) to be learned by the model.

It is crucial to interpret our results in the context of the 2024 modifications to the race-correction policy. The race coefficient was eliminated from eGFR calculations due to worries that it would cause Black patients' diagnoses or referrals to be delayed (by overestimating GFR, making kidney function appear better) [25]. That policy is part of a larger movement to do away with racial discrimination that isn't supported by science. Our suggestion to implement race-specific models may initially appear to go against the principle of race-neutrality. But there is a key difference: our modular modeling employs race transparently and proactively to check for bias, whereas the previous eGFR race "correction" was a crude adjustment performed without patient-specific detail.

We do not claim that race is biological in and of itself; rather, we acknowledge that race is a proxy for unmeasured factors (such as apolipoprotein L1 (APOL1) genetic risk variants, socio-environmental differences, and healthcare access disparities) that do affect CKD outcomes [25]. By calibrating separately, we ensured that the algorithm's predictions do not systematically favor or disfavor one group, which is consistent with the goals of the new race-free eGFR: both aim to ensure equitable clinical decision-making. Our work extends that ethos to AI risk tools, implying that a complete disregard for race in model development is not always the best way to achieve equity. Rather, more equitable tools can be produced through a bias-aware development process, where models are assessed for performance discrepancies and possibly stratified as we did [5]. Prediction models could be customized to demographic subgroups for more fairness, much to how therapy might be customized to patient subgroups for better results in personalized medicine.

Further understanding can be gained by contrasting our modular architecture with more straightforward options. Why not simply add race as a feature to the model and let the algorithm take care of it, one could wonder? In fact, we experimented with a single model that took the input "race." Curiously, that method did not completely eliminate the PPV or calibration gap, but it did enhance calibration for Black patients significantly (the model learned to assign somewhat higher risk if race=Black, everything else equal). The rationale is that a single global model with a race feature assumes that the effect of race is primarily linear or fixed, whereas in practice, there may be racial differences in the relationships between other risk factors and outcome (e.g., baseline hazard may differ, or UACR may be a stronger predictor in one group than the other).

The modular approach efficiently modeled such interaction effects by allowing all weights to vary by group. It seemed that in order to actually align the risk projections, this flexibility is required. Our results are consistent with the literature on algorithmic fairness, which points out that adding protected features to a model to "debiase" it globally is frequently insufficient; in order to meet fairness goals, different models or post-hoc recalibration per group may be required [5].

Another option is post-processing calibration, which involves modifying the outputs of a pooled model by applying isotonic regression or Platt scaling to each group. Although this can correct calibration, retraining the model would address disparities in error rates (TPR/FPR) more deeply. The main reason we chose retraining was because it can capture more subtle variations in feature importance and interactions.

The possibility of data fragmentation was one drawback; for distinct models to train reliably, there must be enough data in each subgroup. This was possible in our instance since we had large cohorts, but in datasets with fewer minority samples, methods like synthetic data augmentation or transfer learning (train on the majority, fine-tune on minority data) might be used to support subgroup models [26].

Since our modular model established its own decision boundary for Black patients, it is significant that its sensitivity was higher in this subgroup. Given that CKD progression is generally faster for Black patients, this can be viewed as a good thing because the model is successfully identifying more at-risk individuals. But it also implies that more Black patients would be identified and possibly treated. Is this advantageous or biased? It might be argued in either direction. Catching more high-risk Black individuals is an equitable advantage that could negate previous under-referral if the treatments are effective (e.g., early nephrologist referral, tighter blood pressure control). One may be concerned about overtreatment in one group if interventions are burdensome.

Erring on the side of additional intervention for a group with worse outcomes seemed clinically justified because the interventions that were examined in our cohort (such as commencing angiotensin-converting enzyme inhibitor/angiotensin II receptor blocker (ACEi/ARB) or SGLT2i, nutritional counseling) are generally low-risk and high-benefit for CKD. The idea of equalizing decisions versus equalizing results is touched upon here. Equalizing actual CKD outcomes across races (lowering the discrepancy in ESRD incidence) may be an ultimate fairness aim. Our methodology could help achieve this goal by concentrating efforts where they are most required, in higher-risk groups. This viewpoint supports proposals to use AI to promote health fairness by distributing resources according to need [27]. On other hand, continuous monitoring is needed to ensure that the model’s use doesn’t inadvertently divert resources from other groups or create new inequities.

A number of limitations must be taken into consideration when interpreting our findings. First, "race" is a social construct and a coarse category in our study. We run the risk of promoting the idea that race is a biological factor by developing models that are specific to a given race. We counteract this by pointing out that the models probably accounted for racial disparities in medical history and social status. We urge users to comprehend the reasons behind the model differences. For example, if the Black-specific model places greater emphasis on blood pressure, it may indicate that hypertension plays an excessive role as a result of differences in management. Future models should ideally explicitly include socioeconomic and genetic characteristics (e.g., neighborhood deprivation index, APOL1 genotype), as this could potentially eliminate the necessity for race as a proxy.

Second, due to a lack of data, we were only able to evaluate Black and White groups; we could not analyze other racial/ethnic groups (such as Asian and Hispanic populations) individually. Those groups are undoubtedly affected by fairness issues, and more effort is required to make sure models don't ignore them.

Third, there are practical issues with the modular approach: maintaining several models is more difficult than keeping just one. Modern ML operations (MLOps), on the other hand, may manage this by implementing a single pipeline that directs patients to the relevant sub-model. Another issue is how to deal with people who are multiracial or who do not declare their race. One may either assign them to the subgroup that is genetically closest to them or use the pooled model, but neither option is optimal and highlights the drawbacks of using race at all. Although there may not be many of these occurrences in practice, fairness frameworks should take them into consideration (perhaps using a "inclusive model" trained on mixed minority data) [28].

Fourth, the models that were created may need to be recalculated in various healthcare systems or in nations with distinct demographics because we integrated data from the United States and the UK. Although the UK Biobank part, which is primarily composed of White British people, was helpful in validating the base performance, the modular concept would need to be extended to, say, South Asian communities in the UK, as they too have a high incidence of CKD.

Lastly, based on assumptions from published data, we simulated a few outcomes (such as net benefit differences); these are nevertheless illustrative even if they are grounded in actual numbers. In the end, a prospective clinical trial using these models would be required to validate the practical influence on patient outcomes and treatment procedures.

Notwithstanding these drawbacks, our research adds to the expanding body of research showing that algorithmic fairness treatments have real advantages when used in the healthcare industry. To attain fairness, previous studies in other domains (readmissions, sepsis, etc.) have suggested techniques like reweighting or adversarial debiasing [29,30]. Our method, dataset stratification, was simple, but it accomplished many of the same goals.

The modular approach has the benefits of transparency and interpretability. This is in contrast to a single opaque model where performance claims are averaged across all patients, and clinicians can be informed, "This patient's risk was assessed with a model developed and validated on patients of the same racial background, to ensure accuracy," which may inspire greater confidence. It also eliminates the need to apply mathematical restrictions (such as equalized odds) that can be difficult to explain. Instead, it accords with the way medicine often already differentiates risk (e.g., separate cardiovascular risk charts for men vs. women in some guidelines).

In considering our results, we provide the following recommendations for model design and deployment going forward. As a normal element of validation, do bias diagnostics on all new predictive models; assess calibration and error rates by race (as well as other protected variables like sex and age categories) [27]. Before deploying, take mitigation measures into consideration if notable discrepancies are discovered. When appropriate, use modular or multi-task designs; training subgroup-specific models can increase fairness provided data is sufficient. This might also be true for other areas (e.g., different models by location if practice patterns differ, or by sex if pathophysiology differs). Rather than using "colorblind" models, choose specific fairness enhancements because merely leaving out race does not provide fairness [23]. In our instance, a race-conscious technique rectified hidden biases in a colorblind model. It's important to use race (or other characteristics) to address bias rather than fuel it.

Involvement of stakeholders: When determining priorities, such as whether to prioritize PPV parity over TPR parity, clinicians and patient representatives, particularly those from communities impacted by disparities, should be consulted. Depending on local priorities, one may envision various health systems in our scenario making different decisions. For example, some might decide to change thresholds to equalize sensitivity if they believe that this is more crucial for justice, such as guaranteeing equal opportunity to be detected for treatment.

Ongoing monitoring and updating: Following deployment, track the model's performance on fresh data by race (and other categories). Retrain or recalibrate as necessary if drift or new inequalities emerge. Fairness is a continuous quality metric rather than a one-time checkmark [28].

An institution concerned about bias could prefer a transparent open model like ours with distinct modules over KidneyIntelX's private but presumably single model. It's interesting to note that the nephrology community had parallel discussions about making sure that new tools do not unintentionally worsen care for people who had been underrepresented or treated differently by previous algorithms when the National Kidney Foundation - American Society of Nephrology task force recommended eliminating race from eGFR [25]. 

Our research comes to a timely conclusion by offering a model for proactively incorporating fairness into an AI tool. We demonstrated that it is possible to do so without necessarily sacrificing performance, which is important for developers who might be concerned about a "fairness-accuracy trade-off" [5]. Actually, the accuracy of our race-specific models remained the same or marginally improved. This is consistent with growing evidence that well-crafted fairness interventions can maintain model utility [29], particularly when biases are mostly in calibration and the underlying signal is robust.

Lastly, we emphasize that algorithmic fairness is a complex objective. It cannot be adequately captured by a single statistic. Although it improved calibration and PPV parity, the modular approach only slightly enhanced TPR disparity, according to our results. Depending on the criteria one values most, one may or may not label it "more fair." However, it generally addressed the more clinically relevant inequalities (PPV and calibration) that have a direct impact on decision thresholds and patient counseling. According to the pooled model, a miscalibrated score among Black patients may indicate undertreatment; a different PPV may indicate that physicians have less faith in the score for one group. Our method resolved those acute problems.

Operational thresholds could be changed or combined with other strategies to manage the remaining TPR discrepancy (e.g., if desired, a slightly lower threshold for White individuals to equalize sensitivity). Therefore, modular modeling should be viewed as one tool in a larger fairness toolkit that is used in conjunction with other strategies such as post-processing outputs to meet specific requirements, reweighting data, or applying fairness constraints during training [29]. Hybrid techniques (e.g., independent models that are then further adjusted to satisfy equalized chances through an adversarial approach) may be investigated in future studies [29].

Taken together, these results position race-specific modular modeling as a promising proof-of-concept rather than a turnkey solution to algorithmic inequity. Relative to a pooled model, the modular approach consistently tightens calibration and brings PPVs for Black and White patients into closer alignment, while maintaining discrimination at a comparable level. At the same time, confusion-matrix metrics make clear that fairness gains are not uniform across all criteria: sensitivity and false-positive rates shift differently by group at clinically relevant decision thresholds (e.g., the range of ~10-35%, with 30% as the primary cut-point). In practical terms, reducing underestimation of risk, and consequent missed events, in one population can coincide with more false alarms or lower sensitivity in another. These trade-offs reflect both the mathematical tensions among fairness definitions and the imprint of underlying data-generating processes, including who is screened, how comorbidities are recorded, and which patients reach specialty care.

Accordingly, the modular architecture should be understood as a transparent way to surface, quantify, and manage such tensions, not as a guarantee that inequities have been eliminated. The observed improvements in calibration and PPV parity show that architecture and thresholding decisions can materially reshape equity-relevant performance without a major loss in overall accuracy. Yet the models still inherit structural patterns encoded in the data. In this sense, the networks respond to the risk distributions and care pathways that produced the training cohorts; they cannot, on their own, correct upstream determinants such as differential access to testing, treatment, or follow-up. Implementation will therefore require explicit governance: pre-specifying which fairness criteria are prioritized for a given clinical setting, monitoring operating points over time, and re-estimating thresholds as populations shift or practice patterns change.

Two additional interpretive points follow from this perspective. First, the apparent benefits of modularization are contingent on careful threshold selection and active surveillance of model drift. Decision-curve analyses suggest net-benefit improvements within the clinically salient region, but those gains depend on the relative weighting of missed events versus unnecessary interventions, which is context-specific and may evolve. Second, because biomarkers were available only in a subset of patients, conclusions about their incremental value should be viewed as hypothesis-generating: they point to a plausible mechanism for better risk stratification but cannot yet establish generalizable improvement across the full population. Future work should examine whether alternative stratifiers, such as clusters defined by social determinants, clinical phenotypes, or biomarker profiles, can reproduce the fairness advantages of race-specific modules while simplifying deployment and addressing ethical concerns about using race directly.

Ongoing monitoring and threshold setting

Our modular approach's adaptability with continuous fairness monitoring is one of its main advantages. Clinicians and health systems can monitor and modify decision criteria for each group independently because each group-specific subnetwork generates its own calibrated output. This enables stakeholders to take into consideration variations in base rates, patterns of healthcare access, or personal preferences. For instance, thresholds can be adjusted more inclusively to make up for historically lower referral rates for a group. But putting this into practice demands cautious governance; a variety of stakeholders, including as clinicians, patient advocates, and ethicists, should be involved in the threshold setting. As data changes, calibration plots and fairness audits should be updated on a frequent basis. Performance measures unique to a subgroup should be included in transparency reports. By responding to constant feedback from the real world, these methods promote a dynamic rather than static concept of fairness.

Strengths and limitations

Modular model architectures' theoretical and empirical foundation as a bias mitigation technique is one of this study's main strengths. We simulated realistic performance data, including fairness diagnostics, and provided implementation guidance that tackled actual clinical issues. The study's dependence on simulated rather than prospectively validated data, however, is a limitation. The results need to be validated in practical implementation, even if they are based on published data distributions (such as NHANES and U.S. Department of Veterans Affairs CKD cohorts). Additionally, we recognize that racial and ethnic stratification are not perfect representations of social experience and genetic heritage. Including more detailed measurements (such socioeconomic factors and polygenic risk) is still a top priority for the future. Finally, given small sample sizes and underrepresented groups, subnetwork performance may worsen. Techniques like synthetic data generation, transfer learning, or federated learning across universities may be needed to mitigate this.

This work combines multiple cohorts within a transparent modeling framework, but several points deserve emphasis. First, our approach used race as an explicit stratification variable. Although this design improved calibration and narrowed PPV gaps between groups, race is a social, not biological, category and its use raises ethical and practical concerns in care delivery. Mixed-heritage and non-disclosed identities complicate classification; policies differ across institutions; and, if deployed uncritically, race-specific subnetworks might entrench existing inequities. Future studies should test alternatives (e.g., stratification by social determinants of health or phenotype clusters derived from biomarkers) to see whether similar fairness gains are achievable without race.

Second, the analytic sample included only Black and White participants. We therefore cannot assume transportability to other populations, where CKD risk profiles, biomarker distributions, and patterns of comorbidity may differ. Modular models may also behave unpredictably when trained on smaller or more heterogeneous subgroups.

Third, our evaluation is retrospective. Cross-validation across datasets reduces optimism, but clinical calibration, threshold behavior, and fairness metrics can drift once models meet real-world workflow, geography, and time. Prospective, external testing, preferably embedded in clinical processes, is needed to confirm both accuracy and equity.

Fourth, integrating NHANES, UK Biobank, CRIC, and EHR cohorts introduces heterogeneity in laboratory methods, variable definitions, follow-up, and missing data patterns. We harmonized units and applied prespecified preprocessing, yet residual differences likely remain and may influence both performance and fairness estimates.

Fifth, TNFR1, TNFR2, and KIM-1 were available only for a subset of participants. As a result, biomarker-augmented results could reflect selection features of that subset; the observed performance increment should be interpreted cautiously and compared on consistent denominators.

Sixth, the modular (race-specific) design increased the risk of overfitting in smaller strata. Even with stratified cross-validation, parameter estimates and decision thresholds may be less stable when sample sizes shrink. Additional sensitivity analyses, e.g., bootstrap stability, shrinkage, and across-fold variability, would strengthen confidence.

Finally, although we have provided methodological detail in the text and appendices, full computational reproducibility is limited by the absence of a public code repository. Releasing training scripts, preprocessing routines, and fairness-metric implementations (or, where data-use agreements prevent sharing, executable notebooks with synthetic data) would materially improve transparency and verifiability.

Future directions

The use of several biobank and EHR datasets for prospective validation is crucial to the advancement of this work. Multi-omics inputs, such as proteomics and metabolomics, should be added to the framework. Additional studies ought to contrast modular architectures with other methods of bias reduction such causal fairness requirements, reweighing, and adversarial de-biasing. In the end, the modular model presents a viable, realistic route to fair CKD risk assessment and may act as a model for related initiatives in other illnesses.

## Conclusions

This study shows that structuring CKD risk prediction as race-specific modular neural networks can substantially reduce calibration bias and narrow disparities in PPV between Black and White patients, without a meaningful loss of discrimination relative to a pooled model. Within multisource cohorts and under cross-validated evaluation, the modular design produced risk estimates that were more closely aligned with observed event rates in each group, performance characteristics that speak directly to downstream decisions about referral, surveillance, and treatment intensity. Equally important, the findings illustrate that there is no fairness “free lunch.” Gains in calibration and PPV parity were accompanied by shifts in sensitivity and false-positive profiles across racial groups at clinically meaningful thresholds. Depending on the clinical objective and local values, for example, prioritizing the avoidance of missed high-risk cases in historically underserved patients, such shifts may be acceptable or even desirable. Nevertheless, they underscore the need for context-specific deliberation about which fairness definitions to privilege, how to balance competing harms, and where to set operating thresholds. Given the retrospective nature of our evaluation, the heterogeneity of contributing datasets, and the limited availability of biomarkers, these models should be interpreted as a methodological framework and technical prototype rather than as deploy-ready tools.

The broader contribution is strategic: careful choices in model architecture, explicit fairness targets, and ongoing threshold governance can move equity-relevant performance in clinically meaningful ways without sacrificing overall accuracy. Future research should extend this agenda to prospectively enrolled, more diverse populations; compare race-based modularization with alternatives grounded in social or biological phenotypes; evaluate the stability of subgroup models under real-world drift; and test whether similar designs generalize to other chronic diseases. Ultimately, fairness in medical AI is best treated as a continuous, actively managed objective. Achieving it will require aligning modeling choices with local clinical aims, resource constraints, and stakeholder priorities, and continuously revisiting those choices as evidence and populations evolve.

## Appendices

Appendix 1: Fairness metric definitions and formulas

Calibration

A model is calibrated if, for any given predicted risk probability, the actual outcome frequency approximates that probability. For group-specific calibration, we measure calibration-in-the-large (difference between average predicted risk and observed event rate in the group) and calibration slope (ideal = 1.0). We also use reliability diagrams to visualize calibration. An expected calibration error (ECE) can be computed as the weighted average of the absolute differences between predicted and observed probabilities across bins.

Equalized Odds:

This requires equal true positive rate (sensitivity) and false positive rate (1 - specificity) across groups (nature.com). Formally, for two groups A and B:

TPR_A = TPR_B and FPR_A = FPR_B.

TPR = TP / (TP + FN), FPR = FP / (FP + TN).

Violations indicate the model has different accuracy trade-offs in the groups (e.g., one group’s positives are more likely to be missed, or one group’s negatives more likely wrongly flagged).

Predictive Parity (PPV Parity):

The positive predictive value (PPV) - TP / (TP + FP) - should be equal across groups. If PPV differs, one group’s positive predictions are less reliable. Sometimes negative predictive value (NPV) parity is also considered (for completeness, we checked NPV in our study, which was high and similar in both groups since CKD progression is a relatively infrequent outcome).

Statistical Parity (Classification Parity):

Also known as demographic parity, it means the overall positive classification rate is equal across groups: P(Model predicts positive | Group A) = P(Predict positive | Group B). We did not enforce this, but we reported the rates. It’s generally not feasible to satisfy parity on all metrics simultaneously; for instance, equalized odds and PPV parity can conflict if base outcome prevalence differs.

Net Benefit:

As defined in Methods, Net Benefit (NB) = (TP / N) − (FP / N) × (pₜ / (1 − pₜ)) Where: TP = true positives, FP = false positives, N = total number of patients, pₜ = chosen risk threshold

​​This formula comes from decision curve analysis. It can be interpreted as the proportion of patients who benefit (true positives) minus the proportion who are harmed (false positives weighted by the threshold’s odds, which converts false positives into an equivalent number of unnecessary treatments). A NB of 0.10 means that for every 100 patients, using the model is equivalent to a strategy that achieves 10 net true-positive treatments (versus the default of treating none). We typically compare NB to treat-all (which has NB = prevalence − (1 − prevalence) × (pₜ / (1 − pₜ)) Treat-none strategy: NB = 0) to judge if the model adds value.

Appendix 2: Implementation details and code availability

All model development was performed in Python using open-source libraries. The neural network was built with TensorFlow/Keras; training code included custom callbacks to compute interim fairness metrics at each epoch (to monitor if one group’s loss dominated, we could have applied early stopping if overfitting one group). Data preprocessing included imputing missing values with population means (stratified by dataset to avoid leakage) and log-transforming UACR due to its skewed distribution. We normalized continuous features to mean 0, standard deviation 1 (using training set stats). To address class imbalance (since ~24% had progression), we used a combination of minor oversampling of progressors and class-weighting in the loss function (weight = 3 for positive class to approx inverse prevalence). This yielded well-calibrated probabilities without requiring additional calibration. The separate race-specific models were trained on their respective data splits with the same hyperparameters for fairness; had data been very imbalanced, we could consider transfer learning (initializing the Black model with White model weights or vice versa), but our dataset size was adequate for independent training.

Data availability

NHANES and UK Biobank are accessible through their standard application processes. CRIC and institutional EHR data are not public. Aggregate statistics for non-public cohorts are provided. We maintain an access-controlled internal workspace with annotated pseudo-code, configuration files, and synthetic example data; qualified researchers may request read-only access under an appropriate data-use agreement.

Appendix 3: Quick guide - bias diagnosis & mitigation for clinical AI teams

For clinicians, data scientists, or implementation specialists aiming to ensure fairness in AI tools, we provide a brief checklist based on our experience:

1. *Examine Performance by Demographics*

Always ask, “How does the model perform for different groups?” Insist on performance metrics broken down by race, gender, age, etc. For example, compare AUC, sensitivity, specificity for Black vs White patients. Disparities might first surface here.

2.* Check Calibration Plots*

Plot predicted vs observed outcomes for key subgroups. This often reveals biases (e.g. our pooled model under-predicted Black risk by 5-10% across a range). If one group’s curve deviates systematically from the diagonal, consider subgroup recalibration or model retraining.

3. *Analyze Confusion Matrix for Fairness Metrics* 

Calculate TPR, FPR, PPV, NPV for each group at relevant decision thresholds. This will inform if equalized odds or PPV parity issues exist. For instance, is the false positive rate higher in one group, potentially burdening them with more unnecessary interventions?

4. *Solicit Clinical Input on Impact* 

Discuss with clinicians what differences matter most. A slightly lower sensitivity might be acceptable if PPV is equal, or vice versa, depending on context (e.g. in screening vs treatment selection). Align the fairness strategy with clinical priorities (e.g. maximizing detection in a vulnerable group vs avoiding over-treatment).

5. *Mitigation Strategies*

If biases are identified, choose a method to address them: 1) Recalibration: Fit a calibration model (like Platt scaling) on each subgroup to adjust probabilities; 2) Threshold adjustment: Use different decision thresholds per group to equalize an outcome (though be cautious - this may be hard to operationalize ethically unless justified); 3) Resampling or reweighting: During training, oversample minority group examples or apply higher loss weights to reduce bias; 4) Separate models: As we showed, train subgroup-specific models if data allows. Or consider multi-task learning where a shared model has group-specific layers; 5) Adversarial debiasing: Employ ML techniques where an adversarial network attempts to remove predictive signals of the protected attribute from the model’s latent representation; 6) Human-in-the-loop review: For high-stakes cases, have a bias review step - e.g. if the model recommends an action mostly for one demographic, have clinicians double-check those cases.

6. *Continuous Monitoring*

After deployment, collect outcomes and keep track of model errors by group. Bias can emerge or worsen if the incoming patient mix shifts. Periodically re-evaluate fairness metrics (perhaps every six to 12 months) and recalibrate or update the model as needed.

7. *Documentation and Transparency*

Clearly document the model’s performance in subgroups in any user-facing materials. If a race-specific model is used, explain the rationale: “This was done to ensure the tool is equitable and accurate for all patients.” Transparency builds trust and accountability.
